# Incorporating preoperative frailty to assist in early prediction of postoperative pneumonia in elderly patients with hip fractures: an externally validated online interpretable machine learning model

**DOI:** 10.1186/s12877-024-05050-w

**Published:** 2024-05-30

**Authors:** Anran Dai, Hao Liu, Po Shen, Yue Feng, Yi Zhong, Mingtao Ma, Yuping Hu, Kaizong Huang, Chen Chen, Huaming Xia, Libo Yan, Yanna Si, Jianjun Zou

**Affiliations:** 1 Department of Pharmacy, Sir Run Run Shaw Hospital, Zhejiang University School of Medicine, Hangzhou, China; 2 School of Basic Medicine and Clinical Pharmacy, China Pharmaceutical University, Nanjing, China; 3 Department of Pharmacy, Shaoxing People’s Hospital, Shaoxing, China; 4 Department of Anesthesiology, Perioperative and Pain Medicine, Nanjing First Hospital, Nanjing Medical University, Nanjing, China; 5 Department of Anesthesiology, The Fourth Affiliated Hospital of Nanjing Medical University, Nanjing Medical University, Nanjing, China; 6 Department of Pharmacy, Nanjing First Hospital, Nanjing Medical University, Nanjing, China; 7 Research and Development Department, Nanjing Xiaheng Network System Co.,Ltd, Nanjing, China; 8 Research and Development Department, Jiangsu Kaiyuan Pharmaceutical Co., Ltd, Nanjing, China

**Keywords:** Postoperative pneumonia, Orthopedic surgery, mFI-5, Risk factor, Catboost, Prediction model

## Abstract

**Background:**

This study aims to implement a validated prediction model and application medium for postoperative pneumonia (POP) in elderly patients with hip fractures in order to facilitate individualized intervention by clinicians.

**Methods:**

Employing clinical data from elderly patients with hip fractures, we derived and externally validated machine learning models for predicting POP. Model derivation utilized a registry from Nanjing First Hospital, and external validation was performed using data from patients at the Fourth Affiliated Hospital of Nanjing Medical University. The derivation cohort was divided into the training set and the testing set. The least absolute shrinkage and selection operator (LASSO) and multivariable logistic regression were used for feature screening. We compared the performance of models to select the optimized model and introduced SHapley Additive exPlanations (SHAP) to interpret the model.

**Results:**

The derivation and validation cohorts comprised 498 and 124 patients, with 14.3% and 10.5% POP rates, respectively. Among these models, Categorical boosting (Catboost) demonstrated superior discrimination ability. AUROC was 0.895 (95%CI: 0.841–0.949) and 0.835 (95%CI: 0.740–0.930) on the training and testing sets, respectively. At external validation, the AUROC amounted to 0.894 (95% CI: 0.821–0.966). The SHAP method showed that CRP, the modified five-item frailty index (mFI-5), and ASA body status were among the top three important predicators of POP.

**Conclusion:**

Our model’s good early prediction ability, combined with the implementation of a network risk calculator based on the Catboost model, was anticipated to effectively distinguish high-risk POP groups, facilitating timely intervention.

**Supplementary Information:**

The online version contains supplementary material available at 10.1186/s12877-024-05050-w.

## Introduction

As the population ages, the incidence of hip fractures continues to rise. It has become a global public health concern [1]. Hip fractures could lead to serious consequences, not primarily due to the rupture itself, but due to the accompanying comorbidities and a range of postoperative complications [2]. Among these, postoperative pneumonia (POP) is one of the most common complications, with an incidence ranging from 4.1–15.2% [3, 4]. Optimizing surgical planning and perioperative management based on preoperative patient status is a promising strategy for early intervention in this complication. Therefore, it is significant to develop a reliable prediction model for early identification and prevention of patients at high risk of POP after hip fracture in the elderly population to improve their postoperative quality of life.

Most of the current studies have focused on the exploration of risk factors for POP. The elderly are prone to multi-organ degeneration, and several comorbidities have been suggested to be independently associated with POP, such as diabetes, respiratory disease, and heart disease [5, 6]. Patients with multiple comorbidities are often in a frail state, with clinical manifestations of reduced physiological reserves, increased vulnerability to death, and increased susceptibility to stress [7]. It has been shown that frail patients have higher postoperative complications and mortality than non-frail patients in orthopedic surgery [8]. Incorporating frailty assessment into routine clinical practice is expected to improve the management of POP in elderly hip surgery patients, but there is insufficient clinical evidence to support it.

It is often difficult to achieve the desired predictive power only through individual predictors and can not give accurate prediction probabilities. Therefore, a tool is needed that can combine multiple predictors and can flexibly capture the direct correlation between predictors and outcome to achieve precise prediction. Large population-based prediction scores for postoperative pulmonary complications have been developed, but they are not specific to pneumonia as an outcome [9, 10]. Zhang et al. [11] and Xiang et al. [12] developed nomograms for predicting POP after hip fracture based on a simplified assessment of the significance of the variables using traditional algorithms. It would be easy to understand but not readily capture the complex relationships between variables. Although the above two nomograms achieved good predictions, they were still not for getting a clinical promotion because they were neither internally nor externally validated, indicating that these good performances may be unreliable, followed by the lack of an online medium for clinical application.

In contrast, the machine learning (ML) approach is considered to be an advanced statistical approach that, in comparison to the “simplified” process of traditional methods, can perform “systematic” inference, making full use of data information. Moreover, as the sample size increases, it can self-learn the updated data and continuously improve the predictive performance. There is still a gap in the application of ML in POP prediction after hip fracture.

Therefore, the main objective of this study was to identify independent risk factors for POP after hip fracture in elderly patients and establish a prediction model based on a ML algorithm to achieve early prediction. In addition, a network risk calculator was also built to provide accurate prediction probabilities to aid clinical decision-making.

## Materials and methods

### Study participants

The derivation cohort consisted of patients with hip fractures who underwent surgical treatment in Nanjing First Hospital (China) between March 2019 and April 2021 and were retrospectively analyzed in this study. Clinical data in the validation cohort were collected from the Fourth Affiliated Hospital of Nanjing Medical University between February 2020 and December 2022. The institutional review boards (IRB) of Nanjing First Hospital (Nanjing, Jiangsu, China) and the Fourth Affiliated Hospital of Nanjing Medical University (Nanjing, Jiangsu, China) approved this study based on the Helsinki declaration (Protocol code: KY20220621-04-KS-01, 20,230,322-k106) and waived the written informed consent requirement owing to the retrospective nature of this study. This study was not concerned with confidential patient information.

### Inclusion and exclusion criteria

Patients aged 65 years or older and hospital admission for femoral neck or trochanteric fracture were included in this study if they underwent total hip replacement or hemiarthroplasty. Conversely, exclusion criteria were patients with (1) pathological fractures; (2) multiple fractures or multiple trauma; (3) conservative treatment; (4) pneumonia that occurred before surgery. Furthermore, some patients, especially those with a history of hip fractures, were deemed ineligible to participate in this study. Finally, some participants were excluded from the study due to missing data on pretreatment features (missing rate > 10%) or the clinical outcome.

### Data collection

All data were obtained from the Surgical Anesthetic Information System and Hospital Information System. After a review of the literature and consultation with clinical experts, the final preoperative available variables for inclusion in the analysis were determined, including demographics (e.g., age, gender, body mass index (BMI)), laboratory measurements (e.g., C-reactive protein (CRP), preoperative hemoglobin), disease history (e.g., hypertension, diabetes mellitus), preoperative incidents (e.g., type of fracture, preoperative length of stay). Frailty was assessed using the modified five-item frailty index (mFI-5), which was based on five variables provided by the National Surgical Quality Improvement Program (NSQIP) [13]. The five variables included congestive heart failure, chronic obstructive pulmonary disease (COPD), diabetes mellitus, hypertension requiring medication, and non-independent functional status (totally or partially dependent functional status) [14, 15]. If a variable was present, it was given 1 point, and the score ranged from 0 to 5 points.

### Outcome

The elemental outcome was pneumonia during the postoperative period before hospital discharge. The criteria for POP diagnosis were based on the NSQIP [16, 17], which required the fulfillment of at least 1 of 2 criteria: (1) the emergence of purulent sputum or a modification in the characteristics of sputum; identification of an organism in a blood culture; pathogen detection in a specimen obtained through trans tracheal aspiration, bronchial brushing, or biopsy; or (2) histopathologic evidence of pneumonia. In addition, they must meet 1 of the following two criteria: (1) the presence of rales or dullness upon percussion during a physical examination of the chest or (2) a chest radiograph that demonstrates new or progressive and persistent infiltrates, consolidation, or cavitation.

### Statistical analysis

The mean and standard deviation were used to describe all normally distributed continuous variables using the t-test method. The median and interquartile range were used for non-normally distributed data, and the Mann-Whitney U-test was employed for analysis. Categorical variables were presented as frequencies (percentages) and assessed through the Chi-square or Fisher’s exact test, as appropriate. A P-value < 0.05 (2-sided) was considered statistically significant. We performed statistical analysis using IBM SPSS software (version 25.0) and R version 4.2.2.

### Data preprocessing

The derivation cohort was divided randomly into two sets: a training set and a testing set, with a ratio of 3:1. The training set was utilized to select features, train the model, and tune hyperparameters. Meanwhile, the testing set was used as an internal validation to assess the reliability and stability of each model. It is common to encounter data that needs to be included in practice. Filling of missing data using K-Nearest Neighbor (KNN) method [18]. Specifically, the missing values were filled in using the KNNImputer module from the “sklearn” package. This module takes into consideration the values of the optimal number of neighbors during the imputation process. This approach allowed us to retain the integrity of the data, and ensure that our analyses were based on full sample size and complete data. Moreover, to prevent data leakage, imputation was performed after splitting the derivation cohort in the training set and testing set. In addition, to ensure consistency in the study, after dividing the training and test sets, all continuous variables were subjected to Z-Score normalization, and categorical variables underwent One-Hot encoding [19, 20]. Python (version 3.10.4) was used for data preprocessing.

### Variable selection

In this study, feature selection was performed on the training set using the least absolute shrinkage and selection operator (LASSO) [21]. The LASSO method uses hyperparameter lambda (λ) to minimize regression coefficients towards zero during the model estimation. This approach excludes many weakly correlated features by assigning their coefficients to zero, while we chose non-zero variables for further analysis. The primary objective of LASSO hyperparametric optimization is to reduce the cost function. Preoperative factors were integrated into the LASSO regression model to evaluate the POP risk in patients before surgery. Lambda was selected from a range of 500 numbers between 0 and 0.5, and ideal hyperparameters that minimized the objective function were identified through 10-fold cross-validation. To prevent errors that a single 10-fold cross-validation could cause, this process was repeated 50 times for each LASSO model. Then, we employed the Variance Inflation Factor (VIF) to evaluate the multicollinearity of the independent variables acquired through LASSO, and factors with VIF > 5 will be excluded [22]. Multivariable logistic regression analysis was performed to determine the variables predicting POP, and the results were expressed as odds ratios (OR) and 95% confidence intervals (95% CI). The prediction model was constructed based on variables with statistical significance (*P* < 0.05). The LASSO was performed with R package glmnet 4.1-3.

### Model development

In this study, we utilized five different ML classifier algorithms to predict POP. We evaluated their performance: logistic regression (LR), random forest classifier (RFC), categorical boosting (Catboost), extreme gradient boosting (XGB), and light gradient boosting machine (LGBM) [23, 24]. We applied the grid search algorithm and 10-fold cross-validation to optimize the hyperparameters for each model. The grid search approach exhaustively investigates all the possible hyperparameter combinations within a specified range to identify the optimal selection. Meanwhile, the 10-fold cross-validation randomly divided the data into ten folds or sections, with nine used for training and one for validation, to evaluate the model’s performance thoroughly. Moreover, the class imbalance was handled by setting class weight to the inverse prevalence of their class [25]. The “sklearn 1.0.2”, “xgboost 1.1.1,” and “xgboost 1.5.1” packages in Python were used to construct all ML models.

### Evaluation and validation

The evaluation of models involved an internal validation using 10-fold cross-validation within the testing set, which aimed to assess the stability of the models. Following this, external validation was carried out to evaluate the generalization capability of the models. The area under the receiver operating characteristic curve (AUROC) and its 95% CI were applied as the primary metric to measure the discriminatory power of the models. The AUROC of 0.5 indicated random guessing, while an AUROC of 1.0 indicated perfect classification. A higher AUROC demonstrated better performance of the model in distinguishing between positive and negative cases. The Delong test assessed the statistical differences between two AUROCs for the five models [26]. The optimal threshold of the prediction probability was selected by the receiver operating characteristic (ROC) curve, and the confusion matrix values such as sensitivity, specificity, accuracy, and F1 value were employed to evaluate the risk stratification ability of the models. Additionally, the area under the precision-recall curve (AUPRC) was utilized to quantify the performance of models, specifically the trade-off between precision and recall at different threshold values of the model’s output score. A higher AUPRC indicated better precision-recall trade, meaning the model effectively identified positive cases while minimizing false positives.

The model calibration was evaluated graphically by plotting the predicted probabilities against observed outcomes. The plot can compute the calibration intercept and slope; the perfect values should be 0 and 1, respectively. The Brier score was also used to measure the accuracy of predicted probabilities of each model, and the value 0 indicated a perfect prediction, while 1 showed an inferior prediction. Based on these performance metrics, we selected the best model.

### Model interpretation

SHapley Additive exPlanations (SHAP) values were calculated using the “SHAP 0.40.0” package in Python, which used a game theoretic approach, to explain the output of ML models [27]. These values provide a metric for assessing the relative importance of a feature to other features, taking into account how that feature impacted the loss function. Moreover, the Shapley values indicate the direction of the relationship between corresponding features and the target. The mean absolute Shapley values were used to quantify the SHAP feature importance. The SHAP bar plot visualizes which features influence the model’s prediction most. In contrast, the SHAP scatter plot helps identify whether a variable positively correlates with the outcome.

## Results

### Patient characteristics

**Fig. 1:**
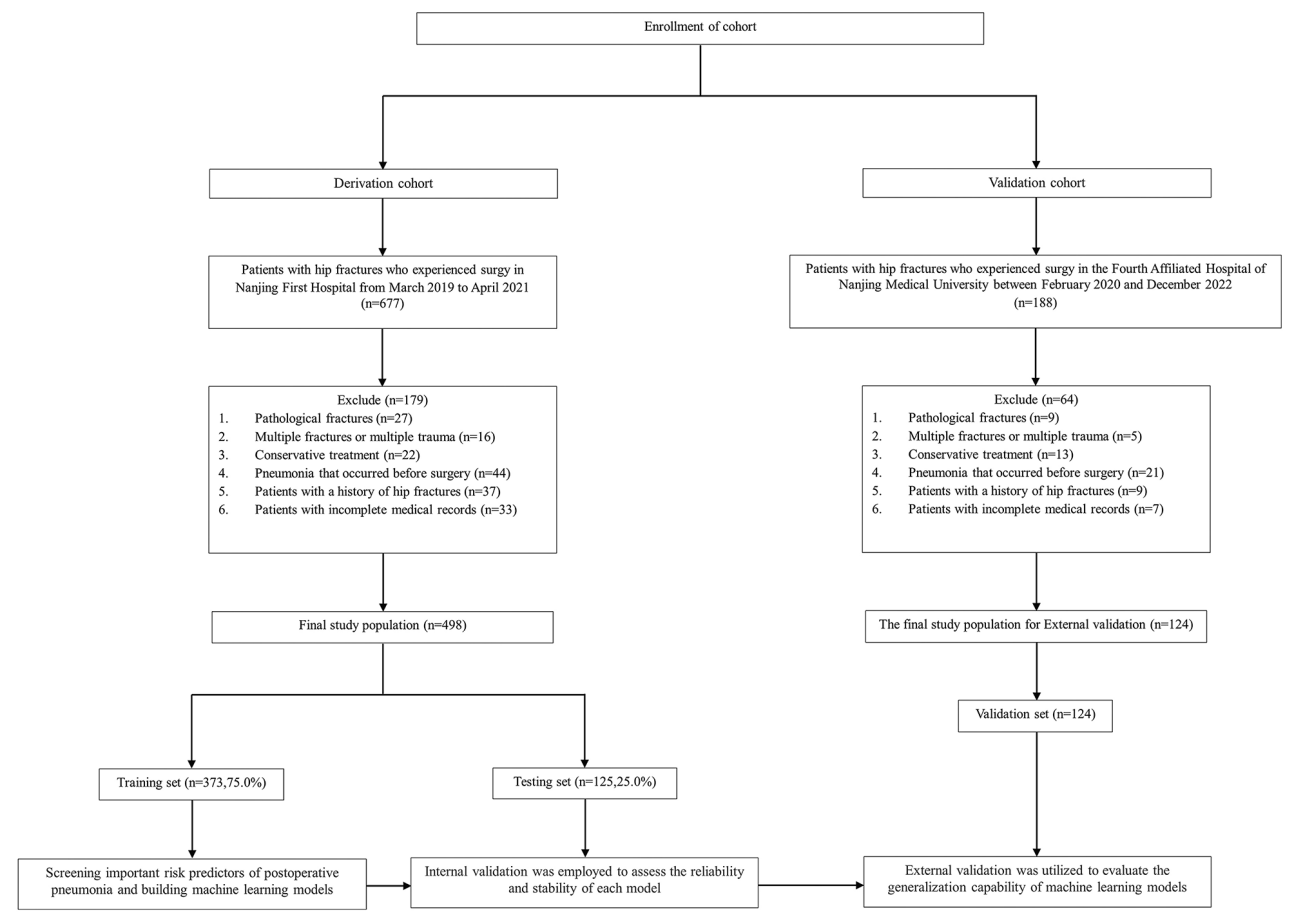
Flow chart of patient enrollment in this study

From March 2019 to April 2021, 498 eligible patients were included in the derivation cohort (Fig. 1). The demographic and clinical characteristics of these patients on admission have been described in Table 1. Among them, 447 and 51 had been diagnosed with femoral neck and trochanteric fractures. Furthermore, 71 patients (14.3%) were diagnosed with POP. Patients with POP were older than those without POP (*P* < 0.001), and there was no statistical difference in gender and BMI. CRP and mFI-5 in patients differed between the two groups (*P* = 0.007 and *P* < 0.001). Chronic obstructive pulmonary disease, heart failure, smoking, preoperative peripheral oxygen saturation (SpO_2_), ASA physical status, and preoperative length of stay differed between patients with and without POP (*P* < 0.05). These patients were randomly assigned to a training set (*n* = 373) or a testing set (*n* = 125), with pneumonia incidence rates of 13.6% and 14.5%, respectively. Demographics and clinical characteristics were almost well-balanced in the two groups (Supplementary Table S1).

**Table 1:** Demographics and Potential Risk Factors of patients in the dataset

Variables	Non-pneumonia (*n* = 427)	Pneumonia (*n* = 71)	*P*-value
Demographics			
Male, n (%)	303 (66.0)	47 (66.2)	1
Age, median (IQR)	77.00 [72.00, 83.00]	83.00 [76.50, 87.00]	< 0.001
BMI, median (IQR)	23.00 [20.00, 25.00]	23.0 0 [21.00, 25.00]	0.423
CRP, median (IQR)	28.50 [9.39, 53.00]	44.40 [22.25, 64.25]	0.007
Cr, median (IQR)	75.80 [53.42, 128.00]	59.00 [46.50, 113.50]	0.08
mFI-5, n (%)			< 0.001
0	109 (25.5)	8 (11.3)	
1	169 (39.6)	23 (32.4)	
2	120 (28.1)	15 (21.1)	
3	25 (5.9)	16 (22.5)	
4	3 (0.7)	9 (12.7)	
5	1 (0.2)	0 (0.0)	
Functional status, n (%)			0.002
Independent	335 (78.5)	43 (60.6)	
Dependent	92 (21.5)	28 (39.4)	
Diabetes mellitus, n (%)	78 (18.3)	45 (63.4)	< 0.001
Chronic obstructive pulmonary disease, n (%)	73 (17.1)	24 (33.8)	0.002
Congestive heart failure, n (%)	10 (2.3)	9 (12.7)	< 0.001
Hypertension, n (%)	228 (53.4)	51 (71.8)	0.004
Preoperative anemia, n (%)	289 (67.7)	52 (73.2)	0.426
Emergency treatment, n (%)	25 (5.9)	3 (4.2)	0.691
Coronary disease, n (%)	176 (41.2)	32 (45.1)	0.632
Atrial fibrillation, n (%)	14 (3.3)	5 (7.0)	0.231
Asthma, n (%)	4 (0.9)	3 (4.2)	0.029
Unexpected cerebrovascular disease, n (%)	135 (31.6)	24 (33.8)	0.819
Smoking, n (%)			0.001
Never smoking	270 (63.2)	29 (40.8)	
Former smoking	71 (16.6)	16 (22.5)	
Current smoking	86 (20.1)	26 (36.6)	
Chronic kidney disease, n (%)	20 (4.7)	5 (7.0)	0.583
Obstructive sleep apnea, n (%)	14 (3.3)	2 (2.8)	1
Mechanical ventilation, n (%)	9 (2.1)	2 (2.8)	0.706
Gastroesophageal reflux, n (%)	21 (4.9)	5 (7.0)	0.456
Myocardial infarction, n (%)	9 (2.1)	2 (2.8)	1
Hepatopathy, n (%)	12 (2.8)	2 (2.8)	1
Preoperative SpO_2_, n (%)			< 0.001
≥ 96%	406 (95.1)	59 (83.1)	
< 96%	21 (4.9)	12 (16.9)	
Fracture type, n (%)			0.008
Femoral neck fracture	390 (91.3)	57 (80.3)	
Trochanteric fracture	37 (8.7)	14 (19.7)	
Type of operation, n (%)			0.018
Total hip replacement	340 (79.6)	47 (66.2)	
Hemiarthroplasty	87 (20.4)	24 (33.8)	
ASA physical status, n (%)			< 0.001
I/II	252 (59.0)	20 (28.3)	
III/IV/V	175 (41.0)	51 (71.8)	
Preoperative length of stay, median (IQR)	3.00 [3.00, 5.00]	5.00 [3.00, 6.00]	< 0.001

BMI, body mass index (calculated as weight in kilograms divided by height in meters squared); CRP, C-reactive Protein; Cr, Creatinine; mFI-5, modified frailty index; SpO_2_, Peripheral capillary oxygen saturation; ASA, American Society of Anesthesiologists

To validate the prediction models from the derivation cohort, an external validation cohort was collected in the Fourth Affiliated Hospital of Nanjing Medical University between February 2020 and December 2022 (Fig. 1). A total of 124 eligible elderly were included in the validation cohort using the same inclusion/exclusion criteria as the derivation cohort. Among them, 13 patients (10.5%) were diagnosed with POP. Supplementary Table S2 provided baseline characteristics of subjects who underwent surgical treatment.

### Feature selection

A few variables had some missing, the specific percentage of missing were listed in Supplementary Table S1, which we filled using the KNN method. In the training set, 24 variables were included in the selection procedure. The LASSO identified eight non-zero coefficient characteristics associated with POP (Supplementary Figure S1). The characteristics included age, CRP, preoperative length of stay, mFI-5, smoking, preoperative SpO_2_, fracture type, and ASA physical status. Furthermore, there was no collinearity among the eight variables (Supplementary Table S3). Multivariable logistics regression analysis was performed for the eight significant variables, and seven independent predictors of POP risk were identified, including age, CRP, preoperative length of stay, mFI-5, smoking, preoperative SpO_2,_ and ASA physical status (Table 2).

**Table 2:** The association of selected variables with pneumonia using multivariate logistic regression in the training set

	Odd Ratio	95% CI	*P*-value
Age	1.05	1-1.1	0.031
CRP	1.01	1-1.02	0.013
Preoperative length of stay	1.3	1.12–1.52	0.03
mFI-5	1.44	1.04–2.02	0.03
Smoking			0.041
Never smoking	1		
Former smoking	1.28	1.11–1.48	0.193
Current smoking	3.34	1.53–7.37	< 0.001
Preoperative SpO_2_			
> 95%	1		
≤ 95%	3.28	1.1–9.34	0.012
ASA physical status			
I/II	1		
III/IV/V	2.98	1.35–6.97	0.031
Fracture type			
Femoral neck fracture	1		
Trochanteric fracture	8.6	0.97-91	0.067

CRP, C-reactive protein; mFI-5, modified five-item frailty index; Preoperative SpO_2_, Preoperative oxygen saturation; ASA, American Society of Anesthesiologists; OR, Odd Ratio; CI, Confidence Interval

### Model performance

**Fig. 2:**
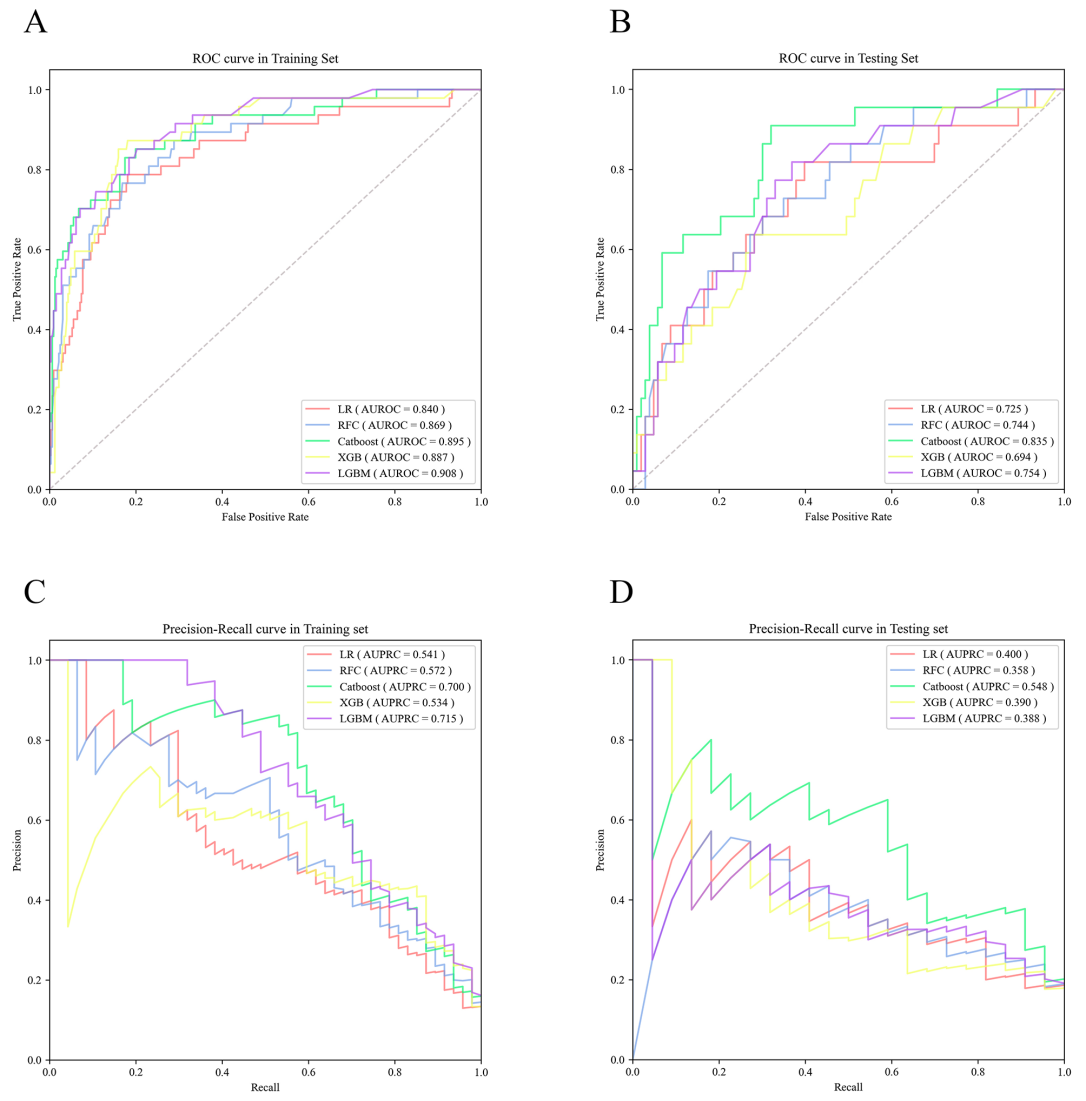
Comparison of AUROC and AUPRC curves among LR, RFC, Catboost, XGB, and LGBM in the training and testing sets. (**A**) AUROC curves of the training set (**B**) AUROC curves of the testing set (**C**) AUPRC curves of the training set (**D**) AUPRC curves of the testing set. AUROC, the area under the receiver operating characteristic; AUPRC, the area under the precision-recall curve; LR, logistic regression; RFC, random forest classifier; Catboost, categorical boosting; XGB, extreme gradient boosting; LGBM, light gradient boosting machine

We constructed five different ML models, including LR, RFC, Catboost, XGB, and LGBM, and evaluated their performance to predict POP occurrence. The best hyperparameter combination for each model was provided in Supplementary Table S4. Figure 2 described their AUROCs and AUPRCs on the training and testing sets. As shown in Fig. 2, on the testing set, the Catboost model yielded the highest AUROC value (median, 0.835; 95%CI: 0.740–0.930) and the highest AUPRC value (median, 0.548; 95%CI: 0.343–0.737). The LGBM model had the next highest AUROC value of 0.754 (95%CI: 0.645–0.864). XGB model had the next highest AUPRC value (median, 0.390; 95%CI: 0.213–0.601). Based on the Delong test, there were statistical differences in the AUROCs between the Catboost model and other models in the testing set (Supplementary Table S5). Additionally, the Youden index of ROC was employed to identify the appropriate threshold for each model. As a result, we obtained the accuracy, sensitivity, specificity, and F1 value of each model under the point, and the results can be shown in Table 3.

**Table 3:** The performance of the five final models under the optimal threshold on the training set and the testing set

	Dataset	Threshold	Accuracy	Sensitivity	Specificity	Brier Score	F1-Value
LR	Training	0.030	0.815	0.787	0.819	0.099	0.517
	Testing		0.760	0.685	0.806	0.152	0.444
RFC	Training	0.038	0.823	0.766	0.831	0.110	0.522
	Testing		0.784	0.595	0.854	0.162	0.426
Catboost	Training	0.151	0.826	0.830	0.825	0.067	0.545
	Testing		0.784	0.786	0.816	0.112	0.509
XGB	Training	0.215	0.842	0.851	0.840	0.100	0.576
	Testing		0.752	0.595	0.816	0.137	0.392
LGBM	Training	0.029	0.804	0.851	0.798	0.095	0.523
	Testing		0.744	0.695	0.786	0.157	0.429

LR, logistic regression; RFC, random forest classifier; Catboost, categorical boosting; XGB, extreme gradient boosting; LGBM, light gradient boosting machine

**Fig. 3:**
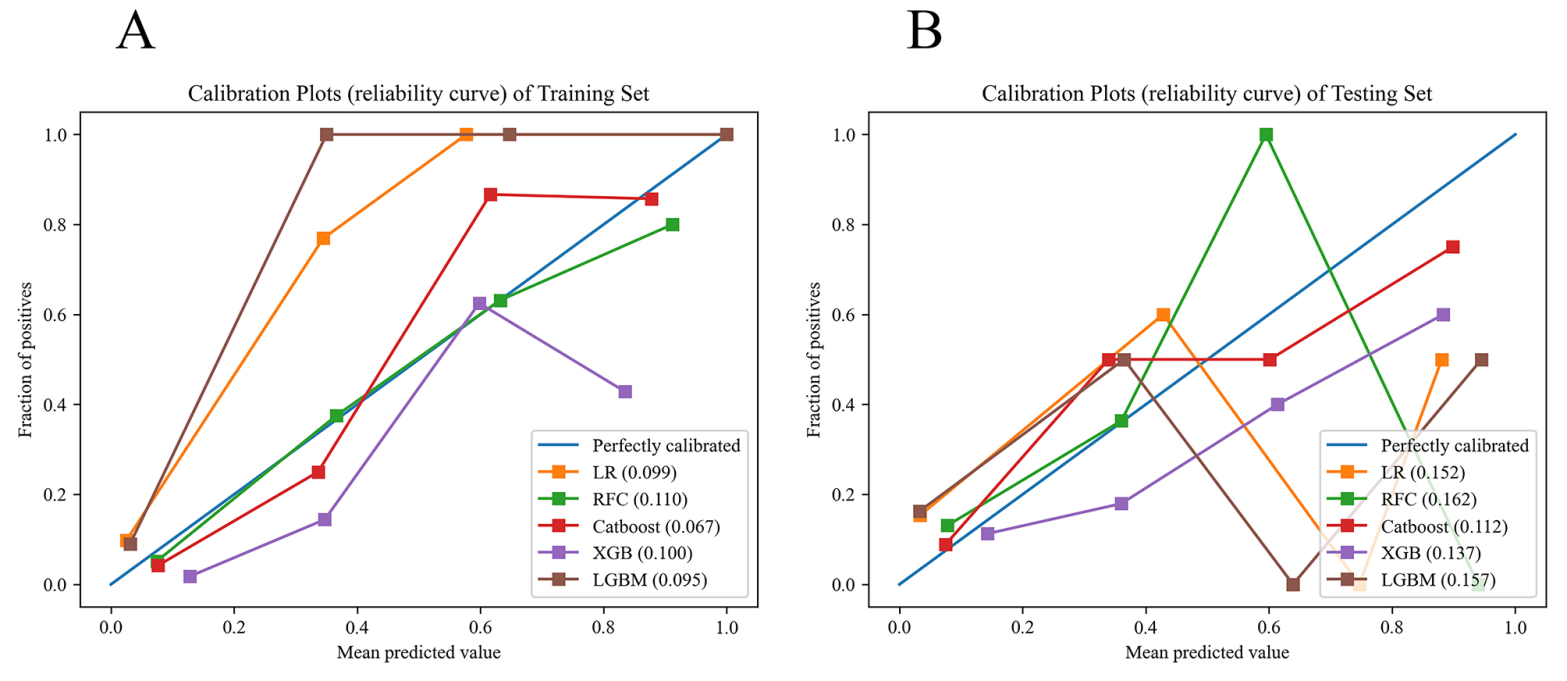
Calibration plots for the probability of pneumonia from the five ML models in the training set (**A**) and the testing set (**B**). LR, logistic regression; RFC, random forest classifier; Catboost, categorical boosting; XGB, extreme gradient boosting; LGBM, light gradient boosting machine

The Catboost model achieved the highest accuracy, sensitivity, and F1 value in predicting POP among ML models on the testing set. The RFC model showed the highest specificity for predicting POP. Significantly, the calibration plot indicated that the Catboost model was positioned closer to the diagonal reference line, yielding the lowest Brier score of 0.112 (Fig. 3).

### External validation

**Fig. 4:**
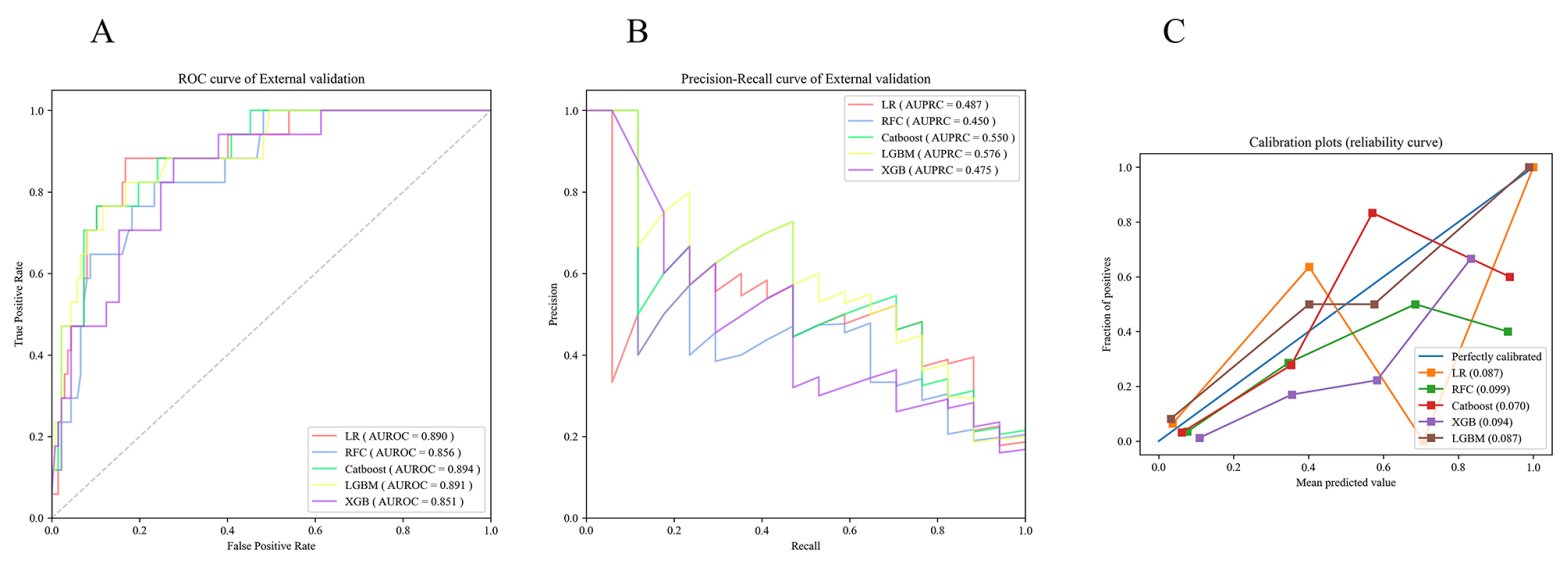
The AUROC curves (**A**), AUPRC curves (**B**), and calibration plots (**C**) from the five ML models in the external validation set. LR, logistic regression; RFC, random forest classifier; Catboost, categorical boosting; XGB, extreme gradient boosting; LGBM, light gradient boosting machine

As shown in Fig. 4, the externally validated AUROC value for the Catboost model was the highest (median, 0.894; 95%CI: 0.821–0.966), followed by the LGBM model (median, 0.891; 95%CI: 0.811–0.970) and the LR model (median, 0.890; 95%CI: 0.814–0.966). The LGBM model yielded the highest AUPRC value (median, 0.576; 95%CI: 0.342–0.780). The Catboost and LR models achieved the next highest AUPRC values of (median, 0.550; 95%CI: 0.320–0.761) and (median, 0.487; 95%CI: 0.269–0.711). The Catboost model had the lowest Brier score of 0.070. Moreover, the Catboost model still showed the highest accuracy of 0.844, specificity of 0.854, and F1-Value of 0.520 among ML models (Table 4).

**Table 4:** The performance of the five final models under the optimal threshold for external validation

	The Threshold	Accuracy	Sensitivity	Specificity	Brier Score	F1-Value
LR	0.030	0.818	0.882	0.810	0.087	0.517
RFC	0.038	0.812	0.765	0.818	0.099	0.473
Catboost	0.151	0.844	0.765	0.854	0.070	0.520
XGB	0.215	0.792	0.706	0.803	0.094	0.429
LGBM	0.029	0.825	0.824	0.825	0.087	0.509

LR, logistic regression; RFC, random forest classifier; Catboost, categorical boosting; XGB, extreme gradient boosting; LGBM, light gradient boosting machine

### Model interpretation

**Fig. 5:**
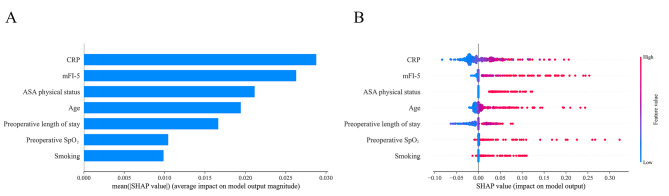
SHAP summary plot for the seven influential variables in the Catboost model. (**A**) The average absolute influence of each factor on the model output magnitude was presented in descending order of feature significance; (**B**) The graph depicted the dot estimate of the Catboost model output, with each dot corresponding to a patient in the dataset. Catboost, categorical boosting; mFI-5, modified five-item frailty index; SpO_2_, Peripheral capillary oxygen saturation; ASA, American Society of Anesthesiologists

The contribution degree of potential risk factors was visualized and ranked by the SHAP method using the Catboost model (Fig. 5), highlighting the most important feature. The results in Fig. 5A demonstrate that CRP, mFI-5, and ASA physical status significantly impacted predicting the outcome. Figure 5B was the scatter plot, in which red and blue dots represented higher and lower values of the features, respectively. The red dots were distributed within the range of positive SHAP values for mFI-5, suggesting that patients with higher scores had a greater risk of developing POP. All predictors were identified as positively correlated with the outcome and considered risk factors.

We also applied this approach to analyze other ML models. As shown in Supplementary Figure S2, preoperative SpO_2_, preoperative length of stay, and smoking were significant variables among the seven factors for these models, indicating that these variables impacted the outcome.

### Construction of the web calculator

**Fig. 6:**
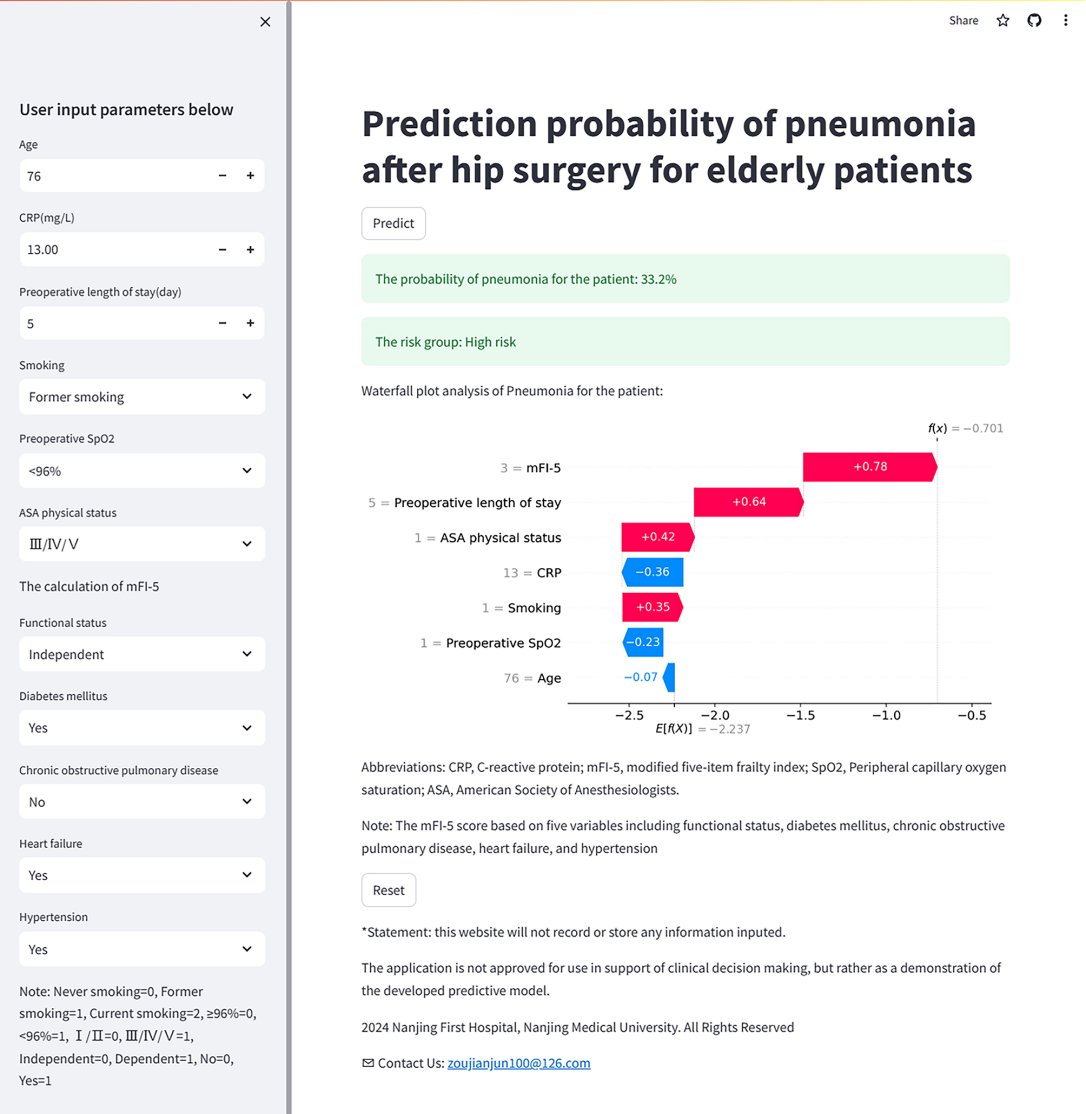
The risk web calculator was designed based on the Catboost model. Catboost, categorical boosting

The Catboost model equations have been integrated into a risk web calculator, accessible at https://prediction-probability-of-pneumonia.streamlit.app/ (Fig. 6). The established web risk calculator could offer clinicians a practical tool to identify high-risk patients for early intervention or a practical demo tool. It also provided research support for the development of medical device software based on the ML algorithm.

## Discussion

Clinicians are often asked to help with preoperative risk assessment and perioperative medical management. In this study, for the first time, we took full advantage of ML to develop and validate an effective early POP prediction model for elderly hip fracture patients by combining seven routinely obtained preoperative variables. We built a web risk calculator to achieve a medium for clinical application.

The Catboost model is considered a powerful ML algorithm that can efficiently handle category-based features and take advantage of ensemble learning to achieve high accuracy predictions [28]. Our study demonstrated that the Catboost model achieved a high AUROC: 0.894 (95%CI: 0.821–0.966) and AUPRC: 0.550 (95%CI: 0.320–0.761) in the external validation set, proving to perform well in the unbalanced datasets. The point was also reflected in the sensitivity (0.765). High sensitivity is crucial for clinical applicability, as failure to correctly identify patients with POP may have serious consequences compared to acceptable interventions for patients without POP.

Another advantage of our model was the establishment of a web risk calculator based on the Catboost algorithm that anyone could access online. The probability of a patient’s risk of POP could be output directly after the predictive characteristics were entered, saving time for manual calculation and greatly increasing the ease of clinical application. Moreover, it is important to combine the accurate prediction probability from a complex model with how to obtain the interpretability of that probability. Therefore, we added corresponding SHAP visual interpretation plots to the calculator output results that support getting the value of each variable’s contribution to the outcome probability. To some extent, this improved clinicians’ recognition of the model results. In addition, these variables were all readily accessible preoperatively, facilitating the realization of early risk assessment and reasonable adjustment of perioperative medical management.

Among the predictive variables, the mFI-5 was simplified from the modified 11-item frailty index (mFI-11), making it easier to utilize in daily clinical practice. And the mFI-5 has been reported to be as effective as the mFI-11 in predicting mortality, postoperative infection, and unplanned 30-day readmission [13]. In a prospective study, frailty has been considered to influence the susceptibility and severity of community-acquired pneumonia in elderly patients [29]. In patients with hip fractures, a high mFI-5 was significantly associated with poor functional recovery, total complications, and serious medical complications (e.g., cardiac arrest, myocardial infarction, and septic shock) [30, 31]. Elderly patients with high mFI-11 who underwent abdominal surgery were also confirmed to have a higher risk of postoperative PPCs [32]. The positive association of mFI-5 with the probability of POP in elderly patients with hip fractures could also be seen in our SHAP summary plots. Besides, although frailty is usually age-related, frailty related to disease still accounts for an important part [33, 34]. In these patients, disease or comorbidities are probably the most significant cause of the decline in physiological reserve.

In addition to the non-modifiable factors of mFI-5, age, and ASA, those potentially modifiable factors (e.g., CRP, SpO_2_ and smoking) may be of greater concern. Firstly, preoperative CRP reflects the inflammatory status of the patient. Although it is a nonspecific marker of systemic inflammation, it has been proven to be a predictive variable of postoperative infection (including pneumonia, surgical site infection, and urinary tract infection) and mortality in hip fracture patients [35, 36]. Our study further confirmed the predictive role of CRP on the occurrence of independent POP infection rather than postoperative overall infection symptoms in elderly patients with hip fractures, with its contribution value to the prediction model ranked first. Secondly, low preoperative SpO_2_ increased the risk of POP, which was consistent with the findings of Russotto, V et al. [37]. SpO_2_ has also been identified as a predictive variable of postoperative respiratory failure and postoperative pulmonary complications [38, 39]. This simple, non-invasive indicator provides early warning for patients with low lung function. Clinicians could take measures such as lung function exercise for early intervention in patients with preoperative SpO_2_ below 96% to reduce the risk of POP [40]. Thirdly, preoperative smoking cessation is strongly recommended for smoking patients, and guidelines have shown that this preventive measure could reduce patients’ perioperative risk, including the occurrence of POP [41, 42].

Patients with delayed surgery have a longer length of bed rest, which may increase the risk of exposure to pro-inflammatory conditions, and reduces the patient’s ability to expel sputum, thereby increasing the risk of POP [43, 44]. Numerous studies and guidelines recommend that elderly patients with hip fractures receive prompt surgical treatment within 48 h or even earlier after admission [45–47]. Our study indicated that preoperative length of stay is positively associated with the risk of POP, which is consistent with most previous studies [48]. However, the fact remains that for some patients in poor health on admission, necessary preoperative examination procedures and interventions may be required. Balancing the patient’s preoperative status with the length of the wait for surgery remains a critical task for clinicians.

There were still some limitations in this study. Firstly, similar to many retrospective studies, some information was reported by patients or their family members, which inevitably had an innate selection or recall bias. Secondly, the data used for model construction were collected based on a single medical center. Although our model has been validated in a recent three-year database of elderly hip fractures at another medical institution, the sample size was small, the number of patients with positive outcomes was even smaller. And those performance metrics focusing on true positives, such as sensitivity, were calculated based on this rather small number of patients. Future validation of our model in larger sample databases is still needed. Thirdly, this study did not include information on intraoperative variables and perioperative antibiotic use in the analysis, and how this information would affect the occurrence of POP still needs to be further explored in the future. However, modeling only by preoperative factors could enable early clinical prediction and guide early intervention. And it is noteworthy that the details of the surgical protocols and medication regimen for the treatment of hip fractures differed between centers, which helps to explain the heterogeneity of the results across studies.

## Conclusion

In this study, CRP, mFI-5, and ASA body status were the top three important predictors of POP. And to our knowledge, this was the first to identify preoperative mFI-5 as an independent risk factor for POP in elderly people with hip fractures. Subsequently, the POP predictive model based on readily available preoperative variables achieved good accuracy and was corroborated by external data. The established web risk calculator would facilitate clinical application to identify high-risk patients for early intervention or specific care.

### Electronic supplementary material

Below is the link to the electronic supplementary material.

Supplementary Material 1

## Data Availability

The original contributions presented in the study are included in the article and additional files. Further data supporting this study’s findings are available from the corresponding author on reasonable request.

## Abbreviations

POP: postoperative pneumonia
mFI-5: the modified 5-item frailty index
ML: machine learning
CRP: C-reactive protein
SpO_2_: peripheral oxygen saturation
EPCO: the European Perioperative Clinical Outcome
KNN: K-Nearest Neighbor
LASSO: the least absolute shrinkage and selection operator
VIF: Variance Inflation Factor
LR: logistic regression
RFC: random forest classifier
Catboost: categorical boosting
XGB: extreme gradient boosting
LGBM: light gradient boosting machine
AUROC: the area under receiver operating characteristic curve
ROC: the receiver operating characteristic
AUPRC: the area under the precision-recall curve
SHAP: SHapley Additive exPlanations

## References

[1] Lonnroos E, Kautiainen H, Karppi P, Huusko T, Hartikainen S, Kiviranta I, Sulkava R (2006). Increased incidence of hip fractures. A population based-study in Finland. Bone.

[2] Lim J. Big Data-Driven determinants of length of stay for patients with hip fracture. Int J Environ Res Public Health. 2020;17. 10.3390/ijerph17144949. 10.3390/ijerph17144949 PMC7400185 32659953

[3] Bohl DD, Sershon RA, Saltzman BM, Darrith B, Della Valle CJ, Incidence (2018). Risk factors, and clinical implications of Pneumonia after surgery for geriatric hip fracture. J Arthroplasty.

[4] Salarbaks AM, Lindeboom R, Nijmeijer W (2020). Pneumonia in hospitalized elderly hip fracture patients: the effects on length of hospital-stay, in-hospital and thirty-day mortality and a search for potential predictors. Injury.

[5] Yu Y, Zheng P (2022). Determination of risk factors of postoperative pneumonia in elderly patients with hip fracture: what can we do?. PLoS ONE.

[6] Tian Y, Zhu Y, Zhang K, Tian M, Qin S, Li X, Zhang Y (2022). Incidence and risk factors for postoperative pneumonia following surgically treated hip fracture in geriatric patients: a retrospective cohort study. J Orthop Surg Res.

[7] Hoogendijk EO, Afilalo J, Ensrud KE, Kowal P, Onder G, Fried LP (2019). Frailty: implications for clinical practice and public health. Lancet.

[8] Traven SA, Reeves RA, Sekar MG, Slone HS, Walton ZJ (2019). New 5-Factor modified Frailty Index predicts morbidity and mortality in primary hip and knee arthroplasty. J Arthroplasty.

[9] Canet J, Gallart L, Gomar C, Paluzie G, Vallès J, Castillo J, Sabaté S, Mazo V, Briones Z, Sanchis J (2010). Prediction of postoperative pulmonary complications in a Population-based Surgical Cohort. Anesthesiology.

[10] Neto AS, da Costa LGV, Hemmes SNT, Canet J, Hedenstierna G, Jaber S, Hiesmayr M, Hollmann MW, Mills GH, Vidal Melo MF (2018). The LAS VEGAS risk score for prediction of postoperative pulmonary complications: an observational study. Eur J Anaesthesiol.

[11] Zhang X, Shen ZL, Duan XZ, Zhou QR, Fan JF, Shen J, Ji F, Tong DK (2022). Postoperative pneumonia in geriatric patients with a hip fracture: incidence, risk factors and a predictive nomogram. Geriatr Orthop Surg Rehabil.

[12] Xiang G, Dong X, Xu T, Feng Y, He Z, Ke C, Xiao J, Weng Y-M (2020). A Nomogram for Prediction of Postoperative Pneumonia Risk in Elderly hip fracture patients. Risk Manage Healthc Policy.

[13] Subramaniam S, Aalberg JJ, Soriano RP, Divino CM (2018). New 5-Factor modified Frailty Index using American College of Surgeons NSQIP Data. J Am Coll Surg.

[14] Yamashita S, Mashima N, Higuchi M, Matsumura N, Hagino K, Kikkawa K, Kohjimoto Y, Hara I (2022). Modified 5-Item Frailty Index score as prognostic marker after radical cystectomy in bladder Cancer. Clin Genitourin Cancer.

[15] Subramaniam S, Aalberg JJ, Soriano RP, Divino CM. New 5-Factor Modified Frailty Index Using American College of Surgeons NSQIP Data. *Journal of the American College of Surgeons* 2018, *226*. 10.1016/j.jamcollsurg.2017.11.005 29155268

[16] Kazaure HS, Martin M, Yoon JK, Wren SM (2014). Long-term results of a postoperative pneumonia prevention program for the inpatient surgical ward. JAMA Surg.

[17] Wren SM, Martin M, Yoon JK, Bech F (2010). Postoperative pneumonia-prevention program for the inpatient surgical ward. J Am Coll Surg.

[18] Altman NS (1992). An introduction to Kernel and Nearest-Neighbor Nonparametric Regression. Am Stat.

[19] Shalabi L, Zyad S, K B. Data Mining: a Preprocessing Engine. J Comput Sci. 2006;2. 10.3844/jcssp.2006.735.739.

[20] Okada S, Ohzeki M, Taguchi S (2019). Efficient partition of integer optimization problems with one-hot encoding. Sci Rep.

[21] Vasquez MM, Hu C, Roe DJ, Chen Z, Halonen M, Guerra S. Least absolute shrinkage and selection operator type methods for the identification of serum biomarkers of overweight and obesity: simulation and application. BMC Med Res Methodol. 2016;16. 10.1186/s12874-016-0254-8. 10.1186/s12874-016-0254-8 PMC5109787 27842498

[22] Slinker BK, Glantz SA (1985). Multiple regression for physiological data analysis: the problem of multicollinearity. Am J Physiology-Regulatory Integr Comp Physiol.

[23] Pedregosa F, Varoquaux G, Gramfort A, Michel V, Thirion B, Grisel O, Blondel M, Prettenhofer P, Weiss R, Dubourg V et al. Scikit-Iearn: Machine learning in python. *Journal of Machine Learning Research* 2011, *12*.

[24] Dorogush AV, Ershov V, Gulin A. CatBoost: gradient boosting with categorical features support. *ArXiv* 2018, *abs/1810.11363*.

[25] Mosley L (2013). A balanced approach to the multi-class imbalance problem.

[26] DeLong ER, DeLong DM, Clarke-Pearson DL (1988). Comparing the areas under two or more correlated receiver operating characteristic curves: a nonparametric approach. Biometrics.

[27] Lundberg SM, Lee S-I. A unified approach to interpreting model predictions. In Proceedings of the Proceedings of the 31st International Conference on Neural Information Processing Systems, Long Beach, California, USA, 2017; pp. 4768–4777.

[28] Hancock JT, Khoshgoftaar TM. CatBoost for big data: an interdisciplinary review. J Big Data. 2020;7. 10.1186/s40537-020-00369-8. 10.1186/s40537-020-00369-8 PMC7610170 33169094

[29] Zhao L-h, Chen J, Zhu R-x (2022). The relationship between frailty and community-acquired pneumonia in older patients. Aging Clin Exp Res.

[30] Inoue T, Misu S, Tanaka T, Kakehi T, Kakiuchi M, Chuman Y, Ono R (2019). Frailty defined by 19 items as a predictor of short-term functional recovery in patients with hip fracture. Injury.

[31] Traven SA, Reeves RA, Althoff AD, Slone HS, Walton ZJ (2019). New five-factor modified Frailty Index predicts morbidity and mortality in geriatric hip fractures. J Orthop Trauma.

[32] Aceto P, Perilli V, Luca E, Schipa C, Calabrese C, Fortunato G, Marusco I, Lai C, Sollazzi LJE, sciences. p. Predictive power of modified frailty index score for pulmonary complications after major abdominal surgery in the elderly: a single centre prospective cohort study. 2021, *25*, 3798–802. 10.26355/eurrev_202105_25947 34109588

[33] Arakawa Martins B, Visvanathan R, Barrie H, Huang CH, Matsushita E, Okada K, Satake S, Uno C, Kuzuya M. Frailty prevalence using Frailty Index, associated factors and level of agreement among frailty tools in a cohort of Japanese older adults. Arch Gerontol Geriatr. 2019;84. 10.1016/j.archger.2019.103908. 10.1016/j.archger.2019.103908 31319367

[34] Angioni D, Macaron T, Takeda C, Sourdet S, Cesari M, Giudici KV, Raffin J, Lu WH, Delrieu J, Touchon J (2020). Can we distinguish Age-related Frailty from Frailty related to diseases? Data from the MAPT Study. J Nutr Health Aging.

[35] Norring-Agerskov D, Bathum L, Pedersen OB, Abrahamsen B, Lauritzen JB, Jorgensen NR, Jorgensen HL (2019). Biochemical markers of inflammation are associated with increased mortality in hip fracture patients: the Bispebjerg hip fracture Biobank. Aging Clin Exp Res.

[36] Cheng X, Liu Y, Wang W, Yan J, Lei X, Wu H, Zhang Y, Zhu Y (2022). Preoperative risk factor analysis and dynamic online Nomogram Development for Early Infections Following Primary Hip Arthroplasty in geriatric patients with hip fracture. Clin Interv Aging.

[37] Russotto V, Sabate S, Canet J, group P (2019). Of the European Society of Anaesthesiology Clinical Trial, N. Development of a prediction model for postoperative pneumonia: a multicentre prospective observational study. Eur J Anaesthesiol.

[38] Fernandez-Bustamante A, Frendl G, Sprung J, Kor DJ, Subramaniam B, Martinez Ruiz R, Lee JW, Henderson WG, Moss A, Mehdiratta N (2017). Postoperative pulmonary complications, early mortality, and Hospital Stay following noncardiothoracic surgery: a Multicenter Study by the Perioperative Research Network Investigators. JAMA Surg.

[39] Canet J, Gallart L, Gomar C, Paluzie G, Vallès J, Castillo J, Sabaté S, Mazo V, Briones Z, Sanchis J (2010). Prediction of postoperative pulmonary complications in a population-based surgical cohort. Anesthesiology.

[40] Qiu QX, Li WJ, Ma XM, Feng XH (2023). Effect of continuous nursing combined with respiratory exercise nursing on pulmonary function of postoperative patients with lung cancer. World J Clin Cases.

[41] Iida H, Kai T, Kuri M, Tanabe K, Nakagawa M, Yamashita C, Yonekura H, Iida M, Fukuda I (2022). A practical guide for perioperative smoking cessation. J Anesth.

[42] Pierre S, Rivera C, Le Maitre B, Ruppert AM, Bouaziz H, Wirth N, Saboye J, Sautet A, Masquelet AC, Tournier JJ (2017). Guidelines on smoking management during the perioperative period. Anaesth Crit Care Pain Med.

[43] Borges FK, Bhandari M, Patel A, Avram V, Guerra-Farfan E, Sigamani A, Umer M, Tiboni M, Adili A, Neary J (2019). Rationale and design of the HIP fracture accelerated surgical TreaTment and care tracK (HIP ATTACK) trial: a protocol for an international randomised controlled trial evaluating early surgery for hip fracture patients. BMJ Open.

[44] Beloosesky Y, Hendel D, Weiss A, Hershkovitz A, Grinblat J, Pirotsky A, Barak V (2007). Cytokines and c-reactive protein production in hip-fracture-operated elderly patients. Journals Gerontol Ser a-Biological Sci Med Sci.

[45] Griffiths R, Babu S, Dixon P, Freeman N, Hurford D, Kelleher E, Moppett I, Ray D, Sahota O, Shields M (2021). Guideline for the management of hip fractures 2020: Guideline by the Association of Anaesthetists. Anaesthesia.

[46] Sayers A, Whitehouse MR, Berstock JR, Harding KA, Kelly MB, Chesser TJ. The association between the day of the week of milestones in the care pathway of patients with hip fracture and 30-day mortality: findings from a prospective national registry - the National Hip Fracture database of England and Wales. BMC Med. 2017;15. 10.1186/s12916-017-0825-5. 10.1186/s12916-017-0825-5 PMC5367007 28343451

[47] Brox WT, Roberts KC, Taksali S, Wright DG, Wixted JJ, Tubb CC, Patt JC, Templeton KJ, Dickman E, Adler RA (2015). The American Academy of Orthopaedic Surgeons evidence-based Guideline on Management of Hip fractures in the Elderly. J Bone Joint Surg Am.

[48] Moja L, Piatti A, Pecoraro V, Ricci C, Virgili G, Salanti G, Germagnoli L, Liberati A, Banfi G (2012). Timing matters in hip fracture surgery: patients operated within 48 hours have better outcomes. A meta-analysis and meta-regression of over 190,000 patients. PLoS ONE.

