# Supplementary material for: Incorporating preoperative frailty to assist in early prediction of postoperative pneumonia in elderly patients with hip fractures: an externally validated online interpretable machine learning model
Source: BMC Geriatr. 2024 May 30;24:472. doi: 10.1186/s12877-024-05050-w (PMC11137973; doi:10.1186/s12877-024-05050-w)

| **Supplementary Table S1.** Demographics and Potential Risk Factors in the training and testing cohort. | | | | |
| --- | --- | --- | --- | --- |
| Variables | Testing set (n=125) | Training set (n=373) | P-value | % missing |
| Demographics |  |  |  |  |
| Male, n (%) | 78 (62.4) | 122 (67.3) | 0.373 | / |
| Age, median (IQR) | 78.00 [72.00, 85.00] | 78.00 [73.00, 83.00] | 0.794 | / |
| BMI, median (IQR) | 22.00 [20.00, 24.00] | 23.00 [20.00, 25.00] | 0.008 | 0.60 |
| CRP, median (IQR) | 37.03 [18.40, 59.80] | 28.20 [7.96, 53.10] | 0.024 | 0.40 |
| Cr, median (IQR) | 83.70 [54.00, 137.00] | 72.00 [50.00, 121.00] | 0.152 | 1.00 |
| mFI-5, n (%) |  |  | 0.541 |  |
| 0 | 32 (25.6) | 85 (22.8) |  | / |
| 1 | 51 (40.8) | 141 (37.8) |  | / |
| 2 | 32 (25.6) | 103 (27.6) |  | / |
| 3 | 7 (5.6) | 34 (9.1) |  | / |
| 4 | 3 (2.4) | 9 (2.4) |  | / |
| 5 | 0 (0.0) | 1 (0.3) |  | / |
| Functional status, n (%) |  |  | 0.881 |  |
| Independent | 96 (86.8) | 282 (75.6) |  | / |
| Dependent | 29 (23.2) | 91 (24.4) |  | / |
| Diabetes mellitus, n (%) | 34 (27.2) | 89 (23.9) | 0.529 | / |
| Chronic obstructive pulmonary disease, n (%) | 23 (18.4) | 74 (19.8) | 0.825 | / |
| Congestive heart failure, n (%) | 3 (2.4) | 16 (4.3) | 0.494 | / |
| Hypertension, n (%) | 70 (56.0) | 209 (56.0) | 1 | / |
| Preoperative anemia, n (%) | 94 (75.2) | 247 (66.2) | 0.079 | 0.80 |
| Emergency treatment, n (%) | 3 (2.4) | 25 (6.7) | 0.113 | 0.40 |
| Coronary disease, n (%) | 56 (44.8) | 152 (40.8) | 0.49 | 0.40 |
| Atrial fibrillation, n (%) | 5 (4.0) | 14 (3.8) | 1 | / |
| Asthma, n (%) | 1 (0.8) | 6 (1.6) | 0.821 | / |
| Unexpected cerebrovascular disease, n (%) | 42 (33.6) | 117 (31.4) | 0.724 | / |
| Smoking, n (%) |  |  | 0.146 |  |
| Never smoking | 69 (55.2) | 230 (61.7) |  | / |
| Former smoking | 29 (23.2) | 58 (15.5) |  | / |
| Current smoking | 27 (21.6) | 85 (22.8) |  | / |
| Chronic kidney disease, n (%) | 7 (5.6) | 18 (4.8) | 0.915 | 2.81 |
| Obstructive sleep apnea, n (%) | 3 (2.4) | 13 (3.5) | 0.762 | 1.00 |
| Mechanical ventilation, n (%) | 3 (2.4) | 8 (2.1) | 1 | 0.61 |
| Gastroesophageal reflux, n (%) | 7 (5.6) | 19 (5.1) | 1 | 1.20 |
| Myocardial infarction, n (%) | 1 (0.8) | 10 (2.7) | 0.375 | 0.80 |
| Hepatopathy, n (%) | 6 (4.8) | 8 (2.1) | 0.214 | 1.41 |
| Preoperative SpO_2_, n (%) |  |  | 0.776 |  |
| ≥96% | 347 (93.0) | 118 (94.3) |  | / |
| <96% | 26 (7.0) | 7 (5.7) |  | / |
| Fracture type, n (%) |  |  | 0.358 |  |
| Femoral neck fracture | 109 (87.2) | 338 (90.6) |  | / |
| Trochanteric fracture | 16 (12.8) | 35 (9.4) |  | / |
| Type of operation, n (%) |  |  | 0.161 |  |
| Total hip replacement | 91 (72.8) | 296 (79.4) |  | / |
| Hemiarthroplasty | 34 (27.2) | 77 (20.6) |  | / |
| ASA physical status, n (%) |  |  | 0.804 |  |
| Ⅰ/Ⅱ | 69 (54.1) | 203 (52.2) |  | / |
| Ⅲ/Ⅳ/Ⅴ | 56 (45.9) | 170 (47.8) |  | / |
| Preoperative length of stay, median (IQR) | 4.00 [3.00, 5.00] | 3.00 [3.00, 5.00] | 0.581 | / |
| Pneumonia, n (%) | 17 (13.6) | 54 (14.5) | 0.924 | / |
| BMI, body mass index (calculated as weight in kilograms divided by height in meters squared); CRP, C-reactive Protein; Cr, Creatinine; mFI-5, modified frailty index; SpO_2_, Peripheral capillary oxygen saturation; ASA, American Society of Anesthesiologists | | | | |

| **Supplementary Table S2.** The potential predictors of patients in the external validation dataset. | | | |
| --- | --- | --- | --- |
| Variable | Non-pneumonia (n=111) | Pneumonia (n=13) | P-value |
| Age, median (IQR) | 70.00 [67.00, 74.00] | 72.00 [66.00, 74.00] | 0.993 |
| CRP, median (IQR) | 13.80 [6.88, 33.61] | 65.81 [45.45, 86.76] | <0.001 |
| Preoperative length of stay, median (IQR) | 2.00 [2.00, 3.00] | 5.00 [4.00, 5.00] | <0.001 |
| mFI-5, n (%) |  |  | <0.001 |
| 0 | 43 (38.7) | 0 (0.0) |  |
| 1 | 39 (35.1) | 2 (15.4) |  |
| 2 | 17 (15.3) | 2 (15.4) |  |
| 3 | 7 (6.3) | 5 (38.5) |  |
| 4 | 5 (4.5) | 3 (23.1) |  |
| 5 | 0 (0.0) | 1 (7.7) |  |
| Functional status, n (%) |  |  | <0.001 |
| Independent | 79 (81.2) | 0 (0.0) |  |
| Dependent | 32 (28.8) | 13 (100.0) |  |
| Diabetes mellitus, n (%) | 26 (23.4) | 7 (53.8) | 0.044 |
| Chronic obstructive pulmonary disease, n (%) | 8 (7.2) | 5 (38.5) | 0.003 |
| Congestive heart failure, n (%) | 7 (6.3) | 3 (25.0) | 0.09 |
| Hypertension, n (%) | 41 (36.9) | 8 (61.5) | 0.157 |
| Smoking, n (%) |  |  | 0.858 |
| Never smoking | 60 (54.1) | 6 (46.2) |  |
| Former smoking | 23 (20.7) | 3 (23.1) |  |
| Current smoking | 28 (25.2) | 4 (30.8) |  |
| Preoperative SpO_2_, n (%) |  |  | <0.001 |
| ≥96% | 95 (85.6) | 0 (0.0) |  |
| <96% | 16 (14.4) | 13 (100.0) |  |
| ASA physical status, n (%) |  |  | 0.001 |
| Ⅰ/Ⅱ | 88 (79.3) | 4 (30.8) |  |
| Ⅲ/Ⅳ/Ⅴ | 23 (20.7) | 9 (69.2) |  |
| CRP, C-reactive protein; mFI-5, modified five-item frailty index; Preoperative SpO_2_, Preoperative oxygen saturation; ASA, American Society of Anesthesiologists. | | | |

| **Supplementary Table S3.** Collinearity analysis of related variables. | |
| --- | --- |
| Variable | VIF |
| Age | 1.204 |
| CRP | 1.046 |
| Preoperative length of stay | 1.066 |
| mFI-5 | 1.140 |
| Smoking | 1.022 |
| Preoperative SpO_2_ | 1.044 |
| Fracture type | 1.077 |
| CRP, C-reactive protein; mFI-5, modified five-item frailty index; Preoperative SpO_2_, Preoperative oxygen saturation; ASA, American Society of Anesthesiologists; VIF, Variance Inflation Factor. | |

| **Supplementary Table S4.** The optimal hyperparameters of the five ML models. | | |
| --- | --- | --- |
| Model | Hyperparameter | Optimal value |
| LR | C | 20 |
|  | Penalty | l2 |
|  | Solver | liblinear |
|  | Class_weight | 1:85,0:15 |
| RFC | N estimators | 40 |
|  | Max depth | 2 |
|  | Min samples leaf | 8 |
|  | Min samples split | 20 |
|  | Class_weight | 1:85,0:15 |
| Catboost | Iterations | 200 |
|  | Od type | 'Iter' |
|  | Od wait | 600 |
|  | Max depth | 4 |
|  | Learning rate | 0.02 |
|  | L2 leaf reg | 12 |
|  | Random seed | 1 |
|  | Metric period | 50 |
|  | Fold len multiplier | 1.2 |
|  | Loss function | 'Logloss' |
|  | Logging level | 'Verbose’ |
|  | Class weights | [85,15] |
| XGB | Max depth | 2 |
|  | Learning rate | 0.01 |
|  | N estimators | 180 |
|  | Min child weight | 8 |
|  | Gamma | 0.1 |
|  | Subsample | 1 |
|  | Colsample bytree | 0.1 |
| LGBM | Boosting type | gbdt |
|  | Objective | binary |
|  | Colsample bytree | 0.6 |
|  | Feature fraction | 0.6 |
|  | Learning rate | 0.01 |
|  | Max depth | 2 |
|  | N estimators | 200 |
|  | Min data in leaf | 4 |
|  | Min child samples | 20 |
|  | Num leaves | 70 |
|  | Class weight | 1:85,0:15 |
| ML, machine learning; LR, logistic regression; RFC, random forest classifier; Catboost, categorical boosting; XGB, extreme gradient boosting; LGBM, light gradient boosting machine. | | |

| **Supplementary Table S5.** Statistical difference of the AUROC of five ML models. | | | | | | |
| --- | --- | --- | --- | --- | --- | --- |
|  |  | LR | RFC | Catboost | XGB | LGBM |
| Training set | LR | \ | 0.023 | <0.001 | 0.008 | <0.001 |
|  | RFC | 0.023 | \ | 0.012 | 0.184 | <0.001 |
|  | Catboost | <0.001 | 0.012 | \ | 0.590 | 0.253 |
|  | XGB | 0.008 | 0.184 | 0.590 | \ | 0.029 |
|  | LGBM | <0.001 | <0.001 | 0.253 | 0.029 | \ |
| Testing set | LR | \ | 0.459 | <0.001 | 0.487 | 0.360 |
|  | RFC | 0.459 | \ | <0.001 | 0.182 | 0.628 |
|  | Catboost | <0.001 | <0.001 | \ | 0.001 | 0.002 |
|  | XGB | 0.487 | 0.182 | 0.001 | \ | 0.035 |
|  | LGBM | 0.360 | 0.628 | 0.002 | 0.035 | \ |
| ML, machine learning; LR, logistic regression; RFC, random forest classifier; Catboost, categorical boosting; XGB, extreme gradient boosting; LGBM, light gradient boosting machine; AUROC, area under the receiver operating characteristic. | | | | | | |

**Supplementary Figure S1.** Demographic and clinical feature selection using the least absolute shrinkage and selection operator (LASSO) binary logistic regression model. (A) LASSO coefficient profiles of the all features. Each pneumonia-related feature’s trajectory was observed in the LASSO coefficient profiles with the changing of the lambda in the LASSO algorithm. (B) The LASSO model's optimal parameter (lambda) was selected via 10-fold cross-validation using minimum criteria. The partial likelihood deviation (binomial deviation) curve was plotted relative to the log (lambda). A virtual vertical line at the optimal value was drawn using one SE of minimum criterion (the 1-SE bar).


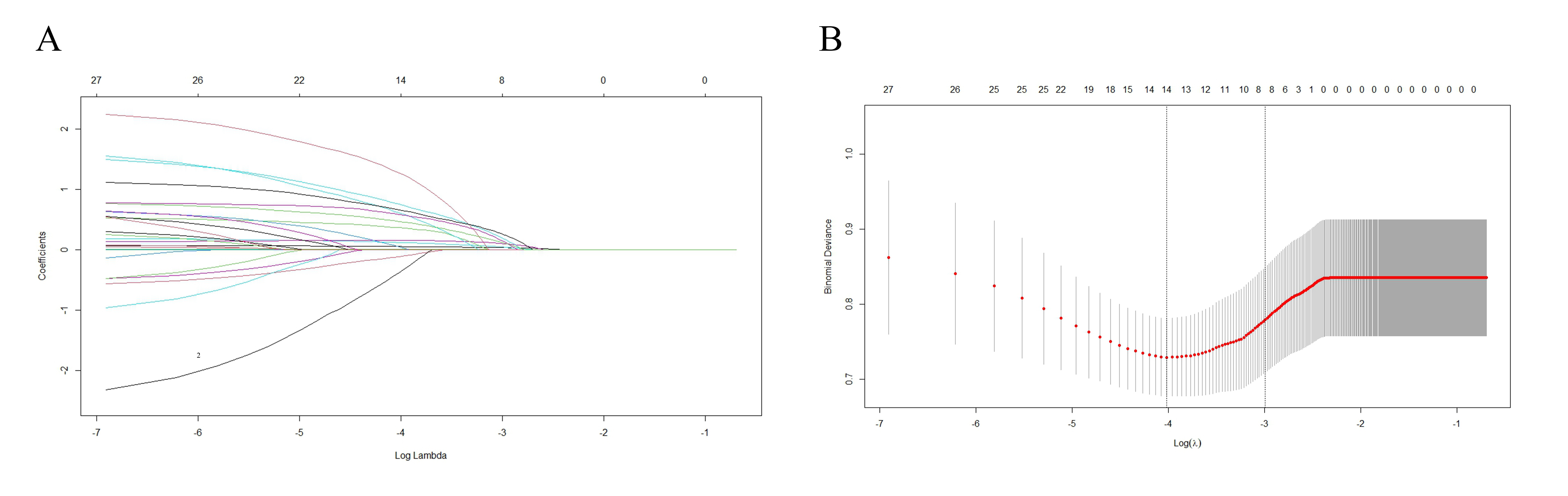


**Supplementary Figure S2.** SHAP summary plot for the seven influential variables in the other predictive models; (A) logistic regression (LR); (B) random forest classifier (RFC); (C) extreme gradient boosting (XGB); (D) light gradient boosting machine (LGBM). mFI-5, modified five-item frailty index; SpO_2_, Peripheral capillary oxygen saturation; ASA, American Society of Anesthesiologists.


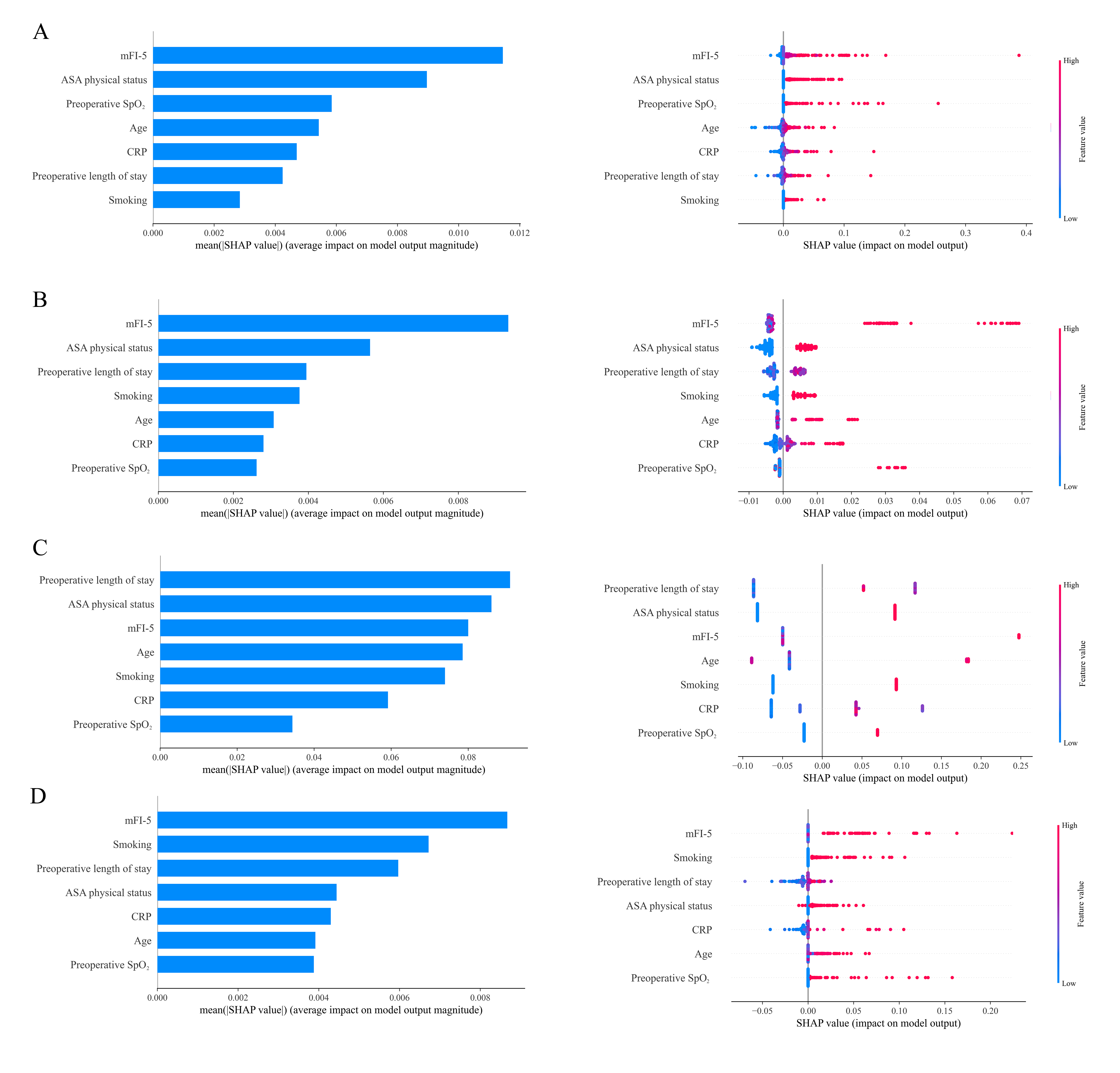

Supplement: Supplementary file 1 — Supplementary Material 1 [file 12877_2024_5050_MOESM1_ESM.docx]
